# Phlegmasia Dolens as a Symptom of Concealed Intra-Abdominal Cancer

**Published:** 1876-04

**Authors:** Henry M. Lyman

**Affiliations:** Professor of Chemistry in Rush Medical College, and one of the Attending Physicians to the Cook County Hospital, Chicago


### PHLEGMASIA DOLENS AS A SYMPTOM OF CONCEALED INTRA-ABDOMINAL CANCER.

By HENRY M. LYMAN, M.D.,

Professor of Chemistry in Rush Medical College, and one of the Attending Physicians to the Cook County Hospital, Chicago.

In his lecture on Phlegmasia Alba Dolens, Trousseau says: "So great, in my opinion, is the semiotic value of phlegmasia in the cancerous cachexia, that I regard this phlegmasia as a sign of the cancerous diathesis as certain as sanguinolent effusion into the serous cavities."

In a lecture on simple chronic ulcer of the stomach, he also says: "Should you, when in doubt as to the nature of an affection of the stomach, should you, when hesitating between chronic gastritis, simple ulcer and cancer, observe a vein become inflamed in the arm or leg, you may dispel your doubt, and pronounce in a positive manner that there is cancer."

This opinion is fortified by the recital of illustrative cases from the experience of the lecturer. To these, which may be read in detail in the published lectures of M. Trousseau, I can add the history of an interesting case which recently came under my observation.

Late in the month of August, 1875, I was asked to prescribe for a gentleman with whom I had maintained a speaking acquaintance during the past ten years. In early childhood he had suffered with morbus coxarius, affecting the left side, and producing permanent deformity of the joint. There had, however, never been any revival of acute symptoms since childhood, and his health had been good for many years. Passing the greater part of youth and early manhood in Europe, his lameness had necessitated a sedentary life, which afforded opportunity for the acquisition of a superior education and the habits of a cultivated society.. Finding his way, at length, to this country, he had settled into the calling of an apothecary, in which he had now reached his forty-ninth year.

Thus introduced, I found my patient sitting in his office one cool day in the early autumn. He had complained since the month of June, of loss of appetite, slight nausea, and a constipated condition of the bowels. His dark eyes seemed unusually bright, and there was about the features a slight degree of sharpness which I had never before remarked. A faint suspicion of impending evil cast a fleeting shadow across my mind, and I made a thorough physical examination which yielded only a negative result. The room was without fire, and as the temperature of the air had been low for several days, I decided in favor of simple functional derangement of the digestive organs, and prescribed accordingly.

About one week later I saw my patient again. He was no better. Had vomited several times; could not drink water without suffering from flatulence and borborygmi. Felt better when the bowels were relaxed. Thought that blue pill and quinine would do him good. I made a second physical examination. Again the results were chiefly negative. The patient was compactly formed and fairly nourished. He experienced no pain in any part of his body. There was nowhere any tenderness on pressure. The posterior third of the tongue was covered with a thin, yellowish coat. The bowels were slightly distended. The pulse was seventy-three per minute, and quite compressible. The tips of the fingers were cool. I could discover no other abnormal symptoms. Examination of the urine exhibited an increase of pigment, but nothing more. I allowed my patient to take a grain of blue pill three times a day, with a saline draught every morning, for about a week, when his evidently failing strength led me to insist upon his retirement from the imperfectly heated atmosphere of his store to the quiet and warmth of his bed.

The symptoms had thus far in no way differed from those which so frequently precede and usher in an autumnal fever; but I was soon convinced that I had to cope with a more serious disease. During the month of

October the vomiting became a daily phenomenon. The intestinal distension was also a source of considerable discomfort, and the intestinal coils were sometimes very clearly defined through the abdominal wall. The bowels were, however, very easily moved, and the patient seemed to derive great relief from three or four evacuations *per diem.* The frequency of the pulse increased to over one hundred beats per minute, but the bodily temperature fell to 96° F., and remained at that point. Before the middle of October I was informed that the vomiting had become stercoraceous ; in fact, the ejected matter had an offensive fœcal odor, and in appearance was closely similar to the pea-soup discharge of typhoid fever. From this time stercoraceous vomiting occurred every few days.

It was now evident that I had to deal with a case of intestinal obstruction. The regions occupied by the liver and spleen began to grow abnormally resonant, like every other portion of the abdominal cavity. It was hardly probable that the stomach could be the seat of the disease, for the stercoraceous vomit was evidently the product of the small intestines ; and the ease with which large injections could be thrown into the colon, and the normal appearance of the stools, pointed to a location at least above the sigmoid flexure of the colon. I felt inclined to believe in the existence of some malignant growth in the omentum which might compress a loop of the intestine, but it was impossible by the most careful palpation to detect anything of the kind.

In this state of perplexity, I one morning discovered that the left leg and thigh of my patient had become swelled, white and shining. Firm pressure over the calf of the leg revealed deep-seated tenderness, and, for the first time, manipulation of the abdomen occasioned slight pain over the internal iliac vein. Remembering the *dicta* of Trousseau, I no longer felt any doubt regarding the nature of the case, and I proceeded to communicate my opinion to an intimate friend of the family. Other physicians were casually consulted (for the patient enjoyed a large medical acquaintance), and their view of the subject was so uniformly cheerful that it would have been difficult for me to adhere to my opinion had I not learned that they had in no instance discovered or taken notice of the existence of phlegmasia dolens.

Well, to make a long story short, matters went on from bad to worse without the slightest benefit from any kind of medication. The unfortunate man suffered no pain, but he could retain no nourishment, and was evidently starving to death. The countenance became Hippocratic, the body was wasted to a skeleton, and finally, in the last days of November, a brief, colliquative diarrhœa terminated his life. As a consequence of this diarrhœa the abdomen became perfectly collapsed, and I made two examinations by external manipulation of every part of the abdominal cavity. I could not discover the slightest indication of tumor or heterologous growth. The liver had retracted into the upper and posterior region of the hypochondriac space. The spleen could not be felt. The spinal column and the aorta were so evident that one gentleman thought it presented an aneurismal enlargement. Death finally occurred without the development of any new symptom, the mind of the patient remaining clear, though indifferent to his condition, until the last.

On the fifth day after decease, an autopsy was performed. It was thought best to examine the abdominal viscera alone. The peritoneal cavity was found empty. The spleen was firm, dark colored, and measured two inches and a half by one inch and a half. I had no means of weighing the viscera, and can only say it was the smallest spleen I ever saw. The liver was contracted and yellow, as if it had undergone acute yellow atrophy. I do not think it would have weighed over, if quite, two pounds. The pancreas presented no unusual appearance. The kidneys were considerably engorged with blood, but were otherwise of normal appearance. The stomach was also healthy. The small intestine was empty. Its parietal capillaries were injected with blood, and the ileum was relaxed to the size of an ordinary colon. The mesenteric glands immediately adjacent to the caput coli were as large as small beans. Elsewhere they were not visible. The ileo-cœcal valve was occupied by a scirrhous growth which deformed the head of the colon and narrowed the passage from the small intestine into the large. I could not detect any other macroscopic lesion. A microscopic examination, by Dr. I. N. Danforth, proved it to be a scirrhous infiltration of the sub-mucous connective tissue, involving the ileo-cœcal valve and the intestinal wall for a distance of half an inch on either side of the valve.

